# In‐house development of a neonatal chest simulation phantom

**DOI:** 10.1120/jacmp.v15i3.4768

**Published:** 2014-05-08

**Authors:** Annemari Groenewald, Willem A. Groenewald

**Affiliations:** ^1^ Vergelegen Oncology Unit Equra Health Somerset West South Africa; ^2^ Department of Medical Imaging and Clinical Oncology Stellenbosch University and Tygerberg Academic Hospital Cape Town South Africa

**Keywords:** neonate, phantom, anatomical equivalence, radiological equivalence, chest radiograph

## Abstract

The aim of the study was to design and construct an anatomical and radiological phantom of a neonatal chest for investigating image quality and patient entrance surface dose (ESD). The density, elemental composition, scatter, attenuation, and absorption characteristics of different possible substitute materials were compared to that of neonatal tissues for radiological equivalence. Availability and cost of possible substitute materials were also considered. The most optimal substitute materials were selected to represent neonatal muscle, bone, healthy or aerated and sick or collapsed lung. For anatomical equivalence, a computed tomography (CT) scan was performed on a neonatal cadaver. Dimensions of different organs and structures were measured with software measuring tools at different window and level settings. Simplifying assumptions, due to machining limitations, were made. The results were used to create scaled drawings, which were used to machine the structures of the phantom. The phantom was assembled in a layer‐by‐layer manner and was validated with region of interest (ROI) analyses. The neonatal chest simulation phantom was an acceptable simulation of a real neonatal chest based on ROI validation, with an overall deviation of 32.5%. The phantom was successfully used in our diagnostic radiology division for ESD and image quality investigations for chest anterior‐posterior (AP) radiographs.

PACS numbers: 87.57.C‐; 87.59.B‐; 41.50.+h

## I. INTRODUCTION

Phantom investigations of dose and image quality relationships give results that can be extrapolated to patients if the phantom used is an acceptable simulation of a patient. This study describes the production of such a neonatal chest simulation phantom, which is radiologically and anatomically similar to a real neonatal chest. For the assessment of radiation dose delivered to a neonate in taking a chest AP X‐ray, a radiologically equivalent simulation phantom would be required. The absorption characteristics of the materials used in the phantom will influence the dosimetry measurement. Radiological equivalence is obtained when the density, elemental composition, attenuation, scatter, and absorption characteristics of a simulating material matches that of real tissue.^(1)^ White et al.^(2)^ published mass density, attenuation, and energy absorption coefficients data for fetal lung, newborn skeletal muscle, and newborn cortical bone and commented that skeletal muscle made up a quarter of the mass of a neonate and could therefore be considered as the main soft body tissue.^(2)^ Clinically applicable visual image quality may also be investigated with a phantom that is anatomically equivalent to a real neonatal chest. The scatter and attenuation characteristics of the materials used in the phantom will influence the obtained image quality. Different attempts on neonatal simulation phantoms are reported in literature.

Brindhaban and Al‐Khalifah^(3)^ used a water‐filled one liter bottle to simulate a 1000 g neonate, which was a crude radiological approximation and in no way anatomically representative of a real neonate. Salez‐Vergara et al.^(4)^ used a PMMA (Perspex, Imperial Chemical Industries, London, UK) phantom. Air gaps were used to represent lungs. Duggan et al.^(5)^ used “white water”^(5)^ and lung‐equivalent inserts. A contrast resolution tool and a line pair resolution gauge were inserted in the phantom for image quality evaluation. Although the phantom offered an improved radiological simulation, it still did not simulate neonatal anatomy accurately. Quantitative image quality could be assessed, but visual image quality could not be evaluated. Akahane et al.^(6)^ constructed a neonatal simulation phantom from rectangular solids. Solid Water and lung phantom materials were used. Its lungs were not symmetrical due to the volume of the heart. Anatomically, the best simulation was provided by the Gammex RMI 610 phantom (Gammex Inc., Middleton, WI).^(7)^ However, the radiological equivalence of the phantom could not be obtained.

It was observed that a suitable anatomical and radiological neonatal chest simulation phantom had not been described in literature. The purpose of this study was not to develop an anthropomorphic phantom, but rather to design and construct a neonatal chest simulation phantom that was an improvement on the phantoms mentioned in literature for ESD and image quality relationship investigation in our diagnostic radiology division. For ESD measurement, a suitable detector (e.g., PTW CONNY II dosimeter; PTW, Freiburg, Germany), is placed on the surface of the proposed phantom, with the phantom set up for an AP chest X‐ray. Such ESD measurements can be used for image quality, both visual and quantitative, versus dose relationship investigation. The proposed phantom would simulate a real neonatal chest anatomically, considering muscle as the main comprising body material, healthy or aerated and sick (hyaline membrane disease or collapsed) lung, and bone (ribs and vertebrae) tissues. It would also be radiologically similar to real neonatal tissues, in terms of tissue density, elemental composition, and attenuation, scatter, and absorption characteristics.

## II. MATERIALS AND METHODS

### A. Radiological equivalence

A variety of possible substitutes, as mentioned by White et al.,^(2)^ ICRU,^(8)^ and Gammex RMI,^(9)^ were considered for neonatal muscle, healthy or aerated lung, sick or collapsed lung, and bone. The different substitute materials were each scored, using the simple scoring system. A mark of 0 to 5 was assigned according to the performance of a possible substitute in each criterion, from 5 for excellent to 0 for not at all. The possible substitute materials were also evaluated according to cost and obtainability, as the details of manufacturing of many possible substitutes were not readily available and most were extremely expensive, hindering its use in the proposed phantom.

The densities and elemental compositions, or mass fractions by weight, were tabulated in literature.^2^, ^8^, ^9^ For agar, a gelatin substitute produced from seaweed, gel mix densities and elemental compositions were not published and were, hence, calculated. The agar gel mix consisted of 4% agar ($C_{12}H_{18}O_{9}$), 10% sucrose ($C_{12}H_{22}O_{11}$), and 86% water ($H_{2}O$). The total attenuation coefficients and incoherent or Compton scatter coefficients were calculated from the mass fractions using the XCOM program.(10) The “mixture rule” (Eq. (1)), was used to calculate the compound mass energy absorption coefficient from its constituent elemental mass fractions and mass energy absorption coefficients.^(11)^

(1) $$\frac{\mu_{en}}{\rho }=\sum_{i}w_{i}{\left(\frac{\mu_{en}}{\rho }\right)}_{i}$$

where $w_{i}$ is the fraction by weight and $\left(\frac{\mu_{en}}{\rho }\right)$ is the mass energy absorption coefficient of the ith element in the compound.^(12)^ Possible substitute data were normalized to that of real neonatal tissues for graphical display (i.e., relative data). A value of 1 would therefore indicate a good match.

### B. Anatomical equivalence

**Figure 1:**
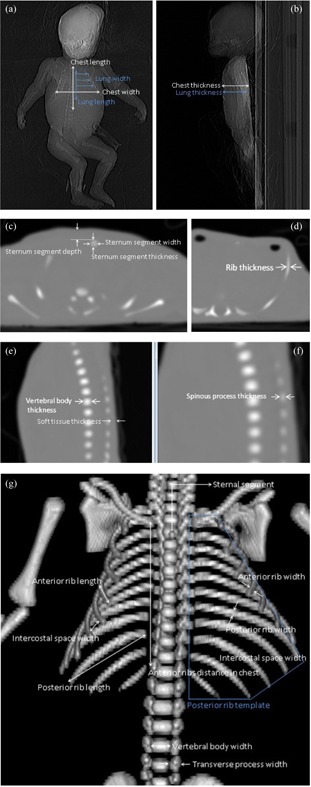
AP CT scout view (a) of neonatal cadaver, lateral CT scout (b) of neonatal cadaver, transverse slice (c) for sternal segment measurements, transverse slice (d) for measuring rib thickness, vertebral thickness measurements (e), a zoomed in version of (e) (f), and reconstructed view for measurements (g).

Health Research Ethics Committee of the University of Stellenbosch approval was obtained (Ethics reference number: N10/12/400) to perform a CT scan on a neonatal cadaver. A Philips Brilliance CT Big Bore configuration scanner (Philips Healthcare, Andover, MA) was used at 120 kV, 30 mA, 400 mm scan length, and 1 mm contiguous slice width. The CT reference was on the sternum of the cadaver. Reconstructions were performed with bone, soft tissue, and lung filters. Measurements were obtained for window width and level settings providing best visualization using the available software tools on the CT scanner, as shown in Fig. 1.

The lungs of the cadaver were not yet air‐filled, therefore the assumption was made that the lungs would have filled the inner cavity of the chest. The sternum was not yet fused and consisted of three bony segments. The sternal segments were assumed to be cuboids for simplification of material machining. It was assumed that the left and right sides of the cadaver would be symmetrical. A simplifying assumption was made that the vertebrae formed a solid column with a length equal to that of the entire chest.

Average sizes and dimensions were calculated from measurements at different locations for the structures incorporated into the neonatal phantom chest. Shapes of structures were also simplified into less rounded and more geometrical shapes. This was done to simplify the machining of the plastics and to take minimum achievable machining limits into account.

### C. Validation of the neonatal chest simulation phantom

**Figure 2:**
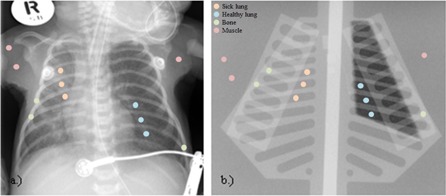
Real neonatal chest x‐ray (a) and X‐ray of neonatal chest simulation phantom (b) used for phantom validation showing schematically the placement of evaluation ROIs.

The average intensities and standard deviations in regions of interest (ROIs) in the healthy and sick lung, bony, and muscle regions of the phantom were compared to those obtained from an X‐ray of a real neonate, as shown schematically in Fig. 2. Averages were calculated from three ROIs in each of the structures at different locations. Both exposures were made at 50 kV, 2 mAs, and inherent filtration at 100 cm focus‐to‐film distance (FFD). The ROIs were sized to fit between the different structures in the phantom and were placed in areas of known material. On the image of the real neonatal chest, ROIs of the same size as in the phantom were used and these were placed in regions that were most homogeneous. This method of validation was also suggested in literature by Duggan et al.^(5)^

## III. RESULTS

### A. Radiological equivalence

**Figure 3:**
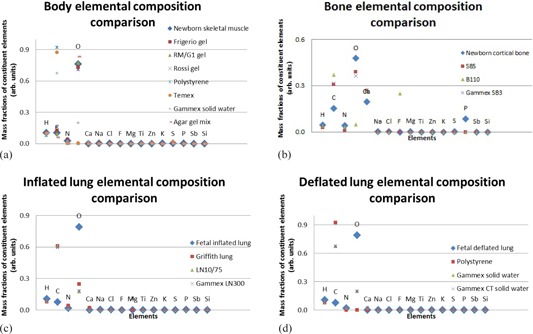
Elemental composition comparison of real neonatal tissues and possible substitute materials including (a) body (muscle), (b) bone, (c) inflated (healthy) lung, and (d) deflated (sick) lung.

**Figure 4:**
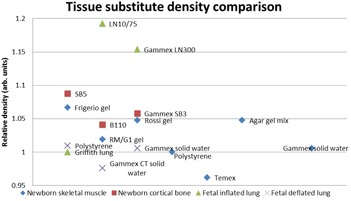
Density comparison for possible substitute materials to real neonatal tissues. A value of 1 indicates a good match.

Figures 3 to 7 were used to determine the 0 to 5 scale scoring of the different possible substitute materials for the different criteria, as recorded in Table 1. The substitute that best matched the corresponding real tissue was awarded a score of 5, the second best option a score of 4, up to a score of 0 if the substitute did not match the real tissue at all. The evaluation is discussed below for each criterion.

**Table 1:** Scoring comparison of different criteria for possible substitute materials

*Tissue Material*	*Substitute Composition*	*Elemental Density*	*Coefficient*	*Total Attenuation*	*Compton Scatter Coefficient*	*Mass Energy Absorption Coefficient*	*Obtainability and Cost*	*Total Score*
Newborn skeletal muscle	Frigerio gel	4	1	3	3	3	‐	14
	RM/G1 gel	4	3	4	3	4	‐	18
	Rossi gel	3	2	2	3	2	‐	12
	Polystyrene	0	4	0	0	0	Local supplier R68.25 per $200\times 100\times 0.1\;{\text{cm}}^{3}$ slab	4
	Temex	0	2	2	0	3	‐	7
	Gammex Solid Water	0	4	3	0	2	Local agent R4902 per $20\times 20\times 1\;{\text{cm}}^{3}$ slab	9
	Agar gel mix	3	2	3	4	3	Local supplier R1700 per kg	15
Newborn cortical bone	SB5	3	1	3	2	3	‐	12
	B110	2	3	3	2	3	‐	13
	Gammex SB3	2	2	3	3	3	Local agent R6726 per $20\times 20\times 1\;{\text{cm}}^{3}$ slab	13
Fetal inflated lung	Griffith lung	3	4	2	3	3		15
	LN10/75	2	2	3	2	2	‐	11
	Gammex LN300	2	3	3	2	2	Local agent R6726 per $20\times 20\times 1\;{\text{cm}}^{3}$ slab	12
Fetal deflated lung	Polystyrene	1	3	2	2	1	Local supplier R68.25 per $200\times 100\times 0.1\;{\text{cm}}^{3}$ slab	9
	Gammex CT Solid Water	3	2	3	3	3	Local agent R5472 per $20\times 20\times 1\;{\text{cm}}^{3}$ slab	14
	Gammex Solid Water	3	3	3	3	3	Local agent R4902 per $20\times 20\times 1\;{\text{cm}}^{3}$ slab	15

Figure 3 shows the elemental composition data graphically. The respective mass fractions, as published in literature,^2^, ^8^, ^9^ are depicted on the y‐axes. The different elements are shown on the x‐axes. Generally, all the possible substitute materials matched the composition of real muscle well, except for polystyrene, Temex, and Gammex Solid Water, which had a very high concentration of carbon and did not contain enough oxygen. Carbon and oxygen differences were also seen in bone, healthy lung, and sick lung substitutes.

The various substitute and real tissue densities, as published in literature,^2^, ^8^, ^9^ are displayed in Fig. 4. In the density comparison, polystyrene proved to be a perfect match to newborn skeletal muscle. For newborn cortical bone substitute B110 scored best. Fetal inflated lung was accurately simulated by Griffith lung. Polystyrene and Gammex Solid Water were the best substitutes for fetal deflated lung.

The results for total attenuation coefficients, calculated with the XCOM program,^(10)^ are recorded in Fig. 5. Polystyrene was not an option as substitute for muscle. The bone substitute results were reasonably comparable. For healthy lung, LN10/75 and Gammex LN300 were comparable. Polystyrene was not an option as a substitute for sick lung. Gammex Solid Water and Gammex CT Solid Water had very similar results.

**Figure 5:**
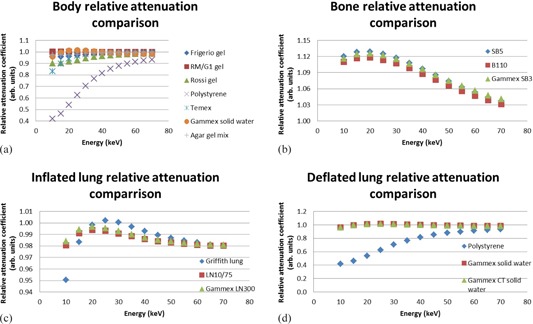
Total attenuation coefficient comparison of real neonatal tissues and possible substitute materials including (a) body (muscle), (b) bone, (c) inflated (healthy) lung, and (d) deflated (sick) lung. A value of 1 indicates a good match.

Figure 6 shows the XCOM calculated Compton scatter coefficient data. When considering Compton scatter coefficients, agar gel mix was the best substitute for newborn skeletal muscle. Temex, polystyrene, and Gammex Solid Water did not show good results. The best substitute for bone was Gammex SB3. Inflated lung was best matched by Griffith lung and polystyrene was the best match to fetal deflated lung.

**Figure 5:**
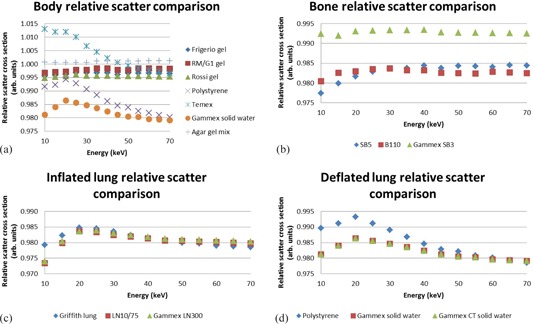
Total attenuation coefficient comparison of real neonatal tissues and possible substitute materials including (a) body (muscle), (b) bone, (c) inflated (healthy) lung, and (d) deflated (sick) lung. A value of 1 indicates a good match.

**Figure 7:**
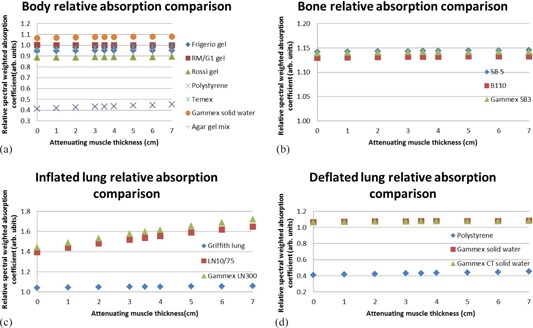
Spectral weighted mass energy absorption coefficient comparison of real neonatal tissues and possible substitute materials including (a) body (muscle), (b) bone, (c) inflated (healthy) lung, and (d) deflated (sick) lung. A value of 1 indicates a good match.

The results for the mass energy absorption coefficient calculation with the “mixture” rule are shown in Fig. 7. RM/G1 gel was found to be an accurate simulator of newborn muscle. The mass energy absorption coefficient results for the bone substitutes were all higher than that of newborn cortical bone. Griffith lung best simulated the mass energy absorption coefficient of the fetal inflated lung. Gammex CT solid water and Gammex Solid Water showed exactly the same results for sick lung absorption coefficient simulation, and polystyrene was not acceptable.

The possible substitute materials were evaluated on obtainability and cost. It was very difficult to find any information on the manufacturing of Frigerio gel, RM/G1 gel, Rossi gel, SB5, B110, Griffith lung, and LN10/75 lung. The basic properties of these substitutes were discussed in the ICRU 44 report.^(8)^ It was not possible to determine the cost of these materials.

Table 1 shows the materials for which manufacturing details and costs were obtainable. It also includes the scoring of the substitutes according to the different criteria as discussed above, based on the results in Figs. 3 to 7. The materials with the highest scores in Table 1 were radiologically the most optimal substitutes for the different real neonatal tissues. These materials matched the density, elemental composition, attenuation, scatter, and absorption characteristics of real neonatal tissues best (i.e., the requirement for radiological equivalence).^(1)^ For neonatal skeletal muscle this was RM/G1 gel, but its manufacturing details were not accessible and so agar gel mix, the second‐best option, was chosen. For this reason, the second‐best option was also selected for fetal inflated lung (i.e., Gammex LN300), although Griffith lung performed best. With newborn cortical bone, both B110 and Gammex SB3 had the same total scores in Table 1. As Gammex SB3 was available locally it was selected as substitute. The best option for fetal deflated lung was Gammex Solid Water.

### B. Anatomical equivalence

**Figure 8:**
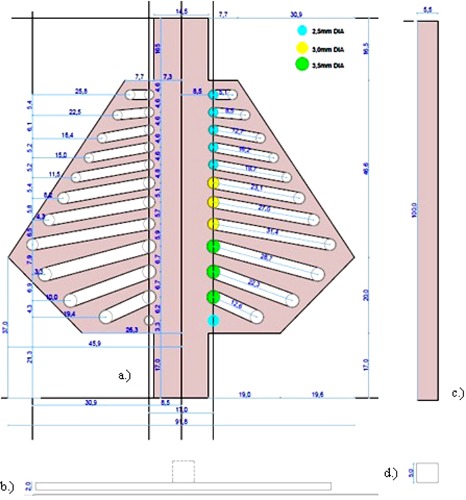
Posterior ribs AP view (a), posterior ribs sectional view (b), vertebral column AP view (c), and vertebral column sectional view (d).

**Figure 9:**
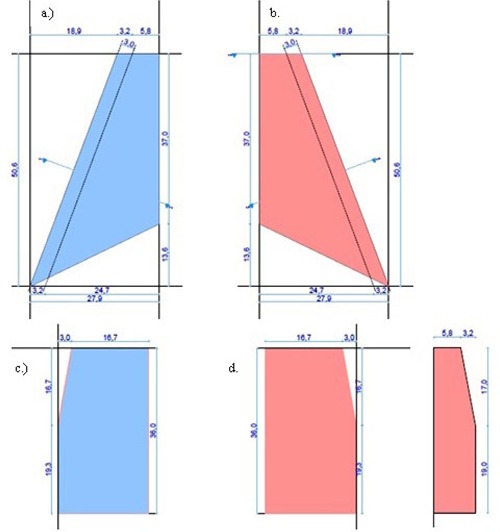
Sick lung AP view (a), healthy lung AP view (b), sick lung sectional view (c), and healthy lung sectional views (d).

**Figure 10:**
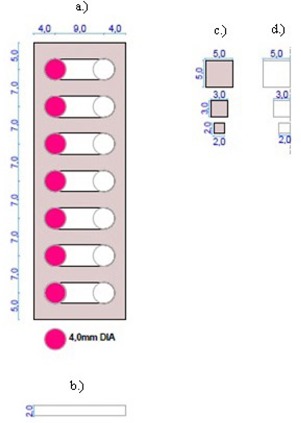
Anterior ribs AP view (a), anterior ribs sectional view (b), sternal blocks AP view (c), and sternal blocks sectional view (d).

From the measurements done, scaled work drawings of the different anatomical structures of interest were prepared (Figs. 8 to 10). These were used for the machining and manufacturing of the posterior ribs, vertebral column, healthy or aerated and sick or collapsed lungs, anterior ribs, and sternal blocks.

The posterior ribs were assumed to be more ridged, geometrical, and with sharper edges and points. Soft tissue gaps were cut from a slab of Gammex SB3 bone‐equivalent material at different diameters and angles. A bony edge was used to account for the increased density of the curl of the ribs in that area. It also gave strength to the structure. The flat section accounted for the transverse processes, which were not actually a solid slab. The vertebral column was simplified as a solid slab. It also accounted for the spinous processes. The lungs were designed to be more ridged, with a good simulation of overall shape. The lateral elongated design accounted for the inferior extension of the lungs posteriorly. The change in thickness of lungs was taken into consideration with angled cuttings. The anterior ribs also had a bony edge to account for the curl of real ribs and to give strength to the structure. These ribs were simplified to have the same length. The anterior ribs structure consisted of a clavicle most superiorly, with seven ribs following, as was seen on the CT scan of the neonatal cadaver. The sternum was not fused in these patients and was assumed to consist of three cuboids, although real notches were more spherical.

**Figure 11:**
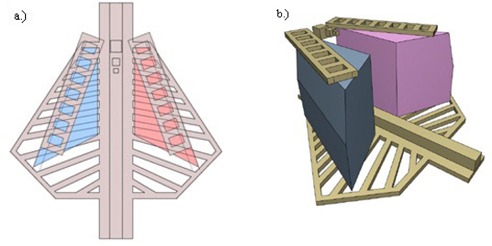
Schematic AP (a) and three‐dimensional (b) representations of the designed neonatal chest simulation phantom.

**Figure 12:**
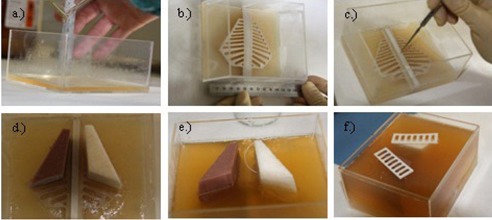
Neonatal chest simulation phantom assembly process: (a) agar gel mix was poured into the Perspex holder; (b) after the first layer set, the posterior ribs were placed centrally in the phantom; (c) the vertebral column was placed on the posterior ribs; (d) the lungs were put in position; (e) placing of the central line; (f) agar gel mix was poured to the level of the lungs and the anterior ribs were placed symmetrically on the lungs.

A schematic AP and three‐dimensional representation of the resultant phantom are shown in Fig. 11. The respective anatomical structures were assembled on a layer‐by‐layer basis in a Perspex holder with the agar gel mixture as muscle substitute material. The process is illustrated in Fig. 12.

### C. Validation of the neonatal chest simulation phantom

The neonatal chest simulation phantom was validated by obtaining the intensities and standard deviations in ROIs placed in muscle, bone, healthy and sick lung areas of images of the neonatal chest simulation phantom and an image of a real neonatal chest. These values were compared. Figure 2 shows these images which were obtained using the same exposure parameters. The results from the ROI analysis are shown in Table 2.

**Table 2:** Phantom validation data

	*Muscle*		*Bone*		*Healthy Lung*		*Sick Lung*	
	*Mean Intensity*	*Standard Deviation*	*Mean Intensity*	*Standard Deviation*	*Mean Intensity*	*Standard Deviation*	*Mean Intensity*	*Standard Deviation*
Real neonatal image	2524.1	74.7	2812.5	125.4	1646.4	78.4	2779.1	85.0
Simulation phantom image	3374.9	25.1	3604.6	24.3	2387.8	50.3	3423.4	23.7
% difference	−33.7		−28.2		−45.0		−23.2	
Average % difference	−32.5							

## IV. DISCUSSION

### A. Radiological equivalence

Agar gel mix was selected as a substitute for neonatal muscle. Agar became a liquid when heated and sets into a firm gel. It was easy to mix and to pour around the anatomical structures of the phantom in layers. The overall results for the three bone substitute materials were very similar. Gammex SB3 was available from a local vendor, at a specified cost. It was chosen as newborn cortical bone substitute material. The creation and price for B110 and SB5 could not be obtained. With fetal inflated or healthy lung, Griffith lung was found to be the best substitute, with Gammex LN300 a close second option. Obtainability and cost were again determining factors. For sick or fetal deflated lung, Gammex Solid Water was the best choice. Gammex CT Solid Water was comparable to Gammex Solid Water and, although both could be obtained from a local representative, Gammex Solid Water was less costly than Gammex CT Solid Water and was, therefore, selected as substitute material.

### B. Anatomical equivalence

Machining restrictions made cutting soft, rounded edges impossible. These resulted in simplifying assumptions which limited the anatomical accuracy of the phantom. It proved to be impossible to machine the anterior curl of the ribs. The vertebral column was assumed solid and a spinal cord was not taken into account. A possible improvement would be the inclusion of a spinal cord by drilling a hole through the solid column structure and filling it with agar gel mix. Vertebrae could also be simulated by separate cuboids and not by a solid column structure. The lungs were mirror images of each other and a manubrium area was not created. The sick lung simulated a completely collapsed lung in the neonatal chest simulation phantom, but in reality, aerated areas often occurred in sick lungs, which could be simulated by the introduction of aerated pockets in the sick lung. The thickness of real lungs also varied over the area of the lungs. More angled cuttings could be made on the simulation lungs to account for thickness variations to a greater extent. Also agar was a growth medium for bacteria and fungi. As a preventative measure, all structures, instruments, and the finished phantom were thoroughly cleaned with Sporekill (ICA International Chemicals (Pty) Ltd, Stellenbosch, South Africa) and the phantom was stored in a refrigerator.

### C. Validation of the neonatal chest simulation phantom

The recorded signal depends on the absorption of the incident radiation on its path through the phantom to the image receptor (i.e., the radiological properties of the phantom). With exposure parameters kept constant, the obtained signal strength (i.e., average intensity) was indicative of the radiological composition of the phantom. A comparison of the mean intensities in ROIs in an image of the phantom to those of a real neonatal chest was therefore an indication of the radiological similarity between the phantom and real neonatal chest. In the case of real neonates, variable density and thickness of the different structures, different over‐ and underlying materials around the structures and nonuniformities in the different organs and structures existed. These were not present in the neonatal chest simulation phantom, which was much more uniform with regard to organ and structure size, shape, density, and composition, because of simplifying geometrical assumptions for easier phantom design and the uniformity of the tissue simulating materials used. The smaller standard deviations in Table 2 for the phantom image compared to the real neonatal chest image confirmed this. These differences accounted for the deviation of the phantom from a real neonatal chest, an overall deviation of 32.5% as Table 2 shows. Although the phantom was not an absolute or accurate simulation of a real neonatal chest, it was an improvement on the phantoms mentioned in literature and was suitable for ESD and image quality relationship investigations.

## V. CONCLUSIONS

A suitable anatomical and radiological neonatal chest simulation phantom was designed in the study. The phantom was an improvement on the phantoms mentioned in literature. Although it was not an anthropomorphic phantom, it was used in our hospital to measure ESD, with a detector placed on the surface of the phantom, and to evaluate image quality. Quantitative image quality was calculated as signal‐to‐noise, signal‐difference‐to‐noise, and contrast‐to‐noise ratios and visual image quality such as the visibility of the sick lung, sternal blocks, central line, posterior ribs, and the transparency of the healthy lung, were assessed by radiographers and medical physicists for different exposure parameters.

## ACKNOWLEDGMENTS

The authors acknowledge iThemba LABS who contributed substantially to the design and development of the neonatal chest simulation phantom.
